# An Anatomy-Informed, Novel Technique for S1 Dorsal Root Ganglion Stimulation Lead Placement

**DOI:** 10.1093/pm/pnac062

**Published:** 2022-04-15

**Authors:** Kenneth B Chapman, Noud van Helmond, Jan Willem Kallewaard, Kris C Vissers, Kiran V Patel, Soriaya Motivala, Jonathan M Hagedorn, Timothy R Deer, David M Dickerson

**Affiliations:** The Spine & Pain Institute of New York, New York, New York, USA; Department of Anesthesiology, NYU Langone Medical Center, New York, New York, USA; Department of Anesthesiology, Northwell Health, New York, New York, USA; Department of Anesthesiology, Pain, and Palliative Medicine, Radboud University Medical Center, Nijmegen, The Netherlands; The Spine & Pain Institute of New York, New York, New York, USA; Department of Anesthesiology, Pain, and Palliative Medicine, Radboud University Medical Center, Nijmegen, The Netherlands; Department of Anesthesiology, Rijnstate Ziekenhuis, Velp, The Netherlands; Department of Anesthesiology, Pain, and Palliative Medicine, Radboud University Medical Center, Nijmegen, The Netherlands; The Spine & Pain Institute of New York, New York, New York, USA; Department of Anesthesiology, NYU Langone Medical Center, New York, New York, USA; Department of Anesthesiology, Northwell Health, New York, New York, USA; Department of Neurosurgery, Northwell Health, New York, New York, USA; iSpine Pain Physicians, Maple Grove, Minnesota, USA; The Spine and Nerve Center of the Virginias, Charleston, West Virginia, USA; Department of Anesthesiology, Critical Care and Pain Medicine, NorthShore University Health System, Evanston, Illinois, USA; Department of Anesthesia & Critical Care, University of Chicago, Chicago, Illinois, USA

**Keywords:** Dorsal Root Ganglion Stimulation, Sacral Nerve, Safety, Neurostimulation, Technique

## Abstract

**Objective:**

A heightened and organized understanding of sacral anatomy could potentially lead to a more effective and safe method of dorsal root ganglion stimulation (DRG-S) lead placement. The aim of this technical note is to describe a standardized access method for S1 DRG-S lead placement.

**Design:**

Technical note.

**Methods:**

The described approach utilizes alignment of the lumbosacral prominence and is measurement-based, allowing for standardized sacral access, even when visualization is suboptimal. The medial-to-lateral needle trajectory is designed to limit interaction with the sensitive neural structures and allows for a more parallel orientation of the lead to the DRG and nerve root.

**Conclusions:**

The described technique potentially improves the safety of S1 DRG-S lead placement. The parallel lead orientation to the DRG may also increase efficacy while lowering energy requirements.

## Introduction

Dorsal root ganglion stimulation (DRG-S) utilizes a shaped electrical field placed over the somata of primary afferent nerve fibers. When compared to dorsal column spinal cord stimulation (SCS) lead placement, DRG-S requires a significantly different technique for electrode placement that continues to evolve. With DRG-S, a curved introducer sheath is used to steer and deploy the lead through the foramen and then over the dorsal root ganglion (DRG), followed by ‘S’ tension loop placement [1].

A developing appreciation for the interplay between DRG-S’ implantable components and methods and the resulting specific anatomical structures in device failure and patient injury is driving technique evolution to improve safety and efficacy. Anchoring the 1-mm DRG-S lead has proven to be an integral step in decreasing lead migration and potentially lead fracture [2]. Additionally, an ipsilateral, paramedian approach for thoracolumbar DRG-S lead placement was described to decrease lead fracture and enable an alternative technique to the wider-angled contralateral approach [3].

Sacral DRG-S leads were initially placed using a retrograde technique, which was supplanted by the posterior transforaminal approach [4, 5]. This more accessible approach increased utilization, and led to the improved understanding of S1 DRG-S’ therapeutic potential, specifically, the multi-dermatomal coverage of neuropathic buttock, leg, and foot pain that DRG-S at S1 provides [6–9]. This becomes apparent when comparing the pivotal ACCURATE study’s use of only a single S1 lead [10], to a 2021 pooled analysis of 756 DRG-S leads which included 248 S1 leads, or 32.8% of the total [2].

Anesthesia for placement of a trial or permanent DRG device has evolved since the early cases were performed. Recognition of the sensitivity of the DRG to mechanical stimuli during placement has led to raised awareness of anesthetic management and the procedural approach [3, 11–13]. Insult to neural structures can cause a neuritis, paresthesia, or weakness, and although commonly self-limiting, longer-term injuries during lead placement in asleep, unmonitored patients have been reported [14, 15]. Sacral neurologic injury in particular has also been reported with sacral nerve stimulation (SNS) from urologic experience [16, 17]. Performing lead placement in the awake patient may reduce such risk, as may the utilization of intra-operative neuromonitoring (IONM) [13, 18].

Given the therapeutic benefits of sacral DRG-S, and the inherent challenges and associated risks of sacral lead placement, the authors detail a novel sacral lead implant technique that relies on fluoroscopic anatomical landmarks and measurements that are generally consistent across the population. Moreover, this technique is designed to limit intra-foraminal instrumentation and the accompanying potential for DRG or nerve root contact by the Tuohy needle or introducer sheath.

## Sacral Anatomy Review

A thorough understanding of sacral anatomy facilitates efficient sacral DRG-S lead and loop placement and reduces multiple common obstacles to placement. The sacrum is formed by the fusion of five progressively smaller sacral vertebrae and their costal elements [19]. The convex shaped triangular sacrum stabilizes the spinal column. The angle where the lordotic lumbar spine meets the sacral promontory is called the lumbosacral angle, which measures roughly 35–40° [20].

Rudimentary spinous processes form the midline sacral crest and on either side of the median sacral crest runs a shallow sacral groove, which gives origin to the multifidus muscle. The floor of the groove is formed by the united lamina of the corresponding vertebrae. Lateral to this lie the intermediate crests, which are formed from the fused articular processes of the sacral vertebrae. At the S1 level the fused joints are the largest, partially obstructing the medial aspect of the S1 posterior sacral foramen (PSF) in the AP view. This ridge narrows caudally with the most inferior aspect forming the sacral cornua; this landmark for the sacrococcygeal hiatus lies just medial to the S4 PSF and is responsible for articulating with the cornua of the coccyx.

### Sacral Canal

The triangular sacral canal measures 27 to 31 mm in width and 12 to 21 mm in AP distance at the S1 level, decreasing in diameter caudally to the sacral hiatus [21–23]. The thecal sac ends at approximately the S2 level.

### Sacral Foramina

The anterior sacral foramen (ASF) at each level communicates with the sacral canal through the intervertebral foramen (IF). The IF is distinct from the ASF and is bordered rostrally and caudally by the pedicles, and the sacral canal medially. The ASF is formed by the fused winged sacral transverse processes, and is a distal extension of the path of the spinal nerve and sometimes the DRG [24]. The PSF is an opening on the dorsal aspect of the ASF, which allow the small dorsal sensory fibers to exit. Each side normally possesses four PSF and ASF, which vary in width, height, and depth. The S1 PSF measures approximately 12 mm × 10 mm and typically can be found caudad to the L5/S1 facet joint. The S2 PSF is slightly smaller, measuring approximately 8 mm × 8 mm [21, 22].

**Figure 1.**
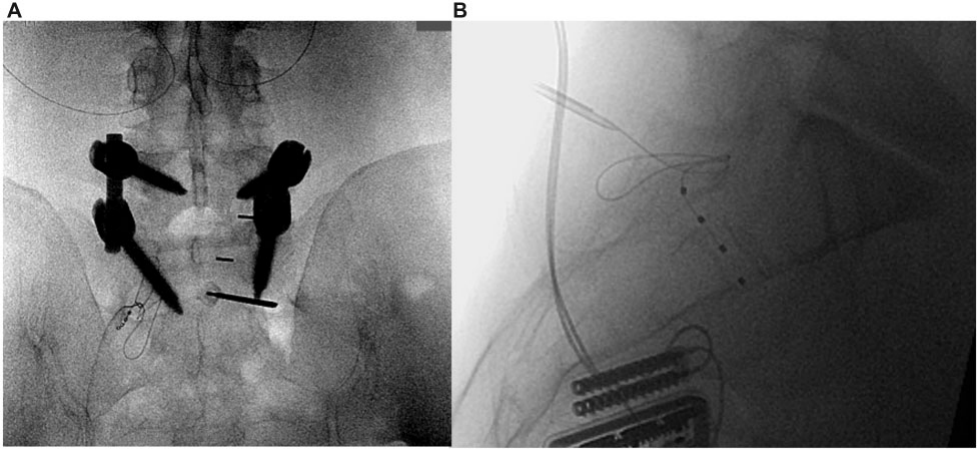
(**A**) Anterior-posterior view of S1 lead placement for failed back surgery syndrome with partially obscured foramen. Medial to lateral access allows the lead to follow the path of the dorsal root ganglion and nerve root. (**B**): Lateral image of the S1 lead placed in a patient as a salvage trial of dorsal root ganglion stimulation at T12 and S1 for failed back surgery syndrome. The remnant of the S1-2 disc can be seen adjacent to the S1 lead and serves as a landmark for placement.

The S1 and S2 ASF are relatively equal in size measuring approximately 13 mm in diameter [21–23]. The rostral border of the S1 PSF lies approximately 2.5 cm from the superior margin of the sacrum, 2 cm from midline, and 2.5 cm from the posterior superior iliac spine [25, 26]. When aligned, the approximate boundaries of the ASF are 6 mm superior, 10 mm lateral, 3 mm inferior, and 3 mm medial to the corresponding margins of the PSF. In the lateral fluoroscopic view, the transverse ridge, a remnant of the fused intervertebral disc, approximates the level of the lower foramen at the S1 and S2 levels—see Figure 1.

### Sacral Nerves and DRG

The ASF fans out inferior-laterally and when measured from the midline of the sacral canal, the DRG and nerve root travels in a lateral oblique angle approximately 15° from the sagittal plane and 28°ventrally, and is accompanied by the foraminal vessels [23, 27].

**Figure 2.**
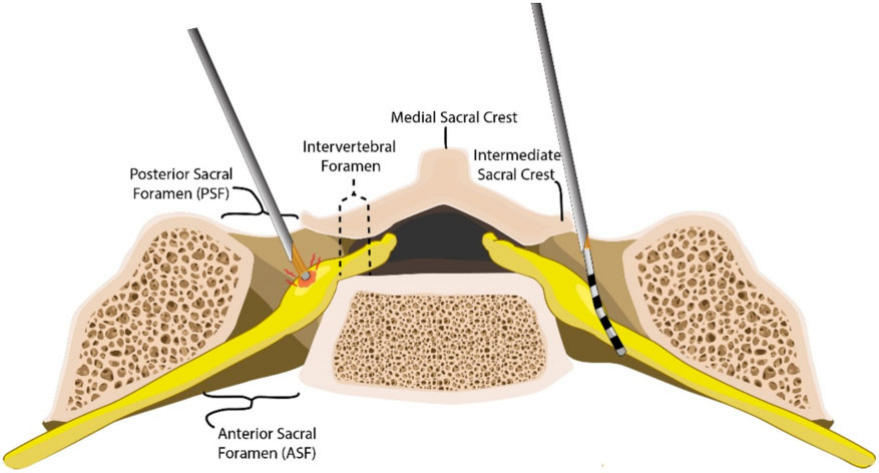
Axial view of the S1 level with (**A**) oblique vs (**B**) the described AP, medial to lateral DRG-S needle placement approach. Note the angle of entry facilitates lead placement over the DRG in the trajectory of the Tuohy needle, while keeping the needle and introducer away from the DRG itself.

The S1 DRG measures roughly 13 mm long x 6 mm wide, compromising nearly 60–70% of the foramen [23]. It lies at or near the intervertebral foramen, with 55–60% of S1 DRG found within the foramen and 40–45% within the sacral canal [28]. These anatomical nuances place the DRG directly in line with the needle trajectory with the commonly used ipsilateral oblique fluoroscopic view for DRG-S approach [29, 30]—see Figure 2. Additionally, when this approach is utilized, the lead is directed medially, likely to deflect off the vertebral body, and approach the DRG in a perpendicular orientation, with only the distal contacts lying adjacent to the laterally spanning DRG and nerve root.

As such, caution must be taken to avoid injuring the DRG when placing S1 leads. The Tuohy needle and/or introducer sheath can cause blunt trauma to the DRG’s sensitive small fiber neurons. If this occurs, symptoms range from a transient paresthesia, as seen with a spinal needle contacting the DRG during transforaminal injections, to more severe symptoms that may take longer to resolve.

## Novel Sacral DRG-S Lead Placement Technique

### Visualization

**Figure 3.**
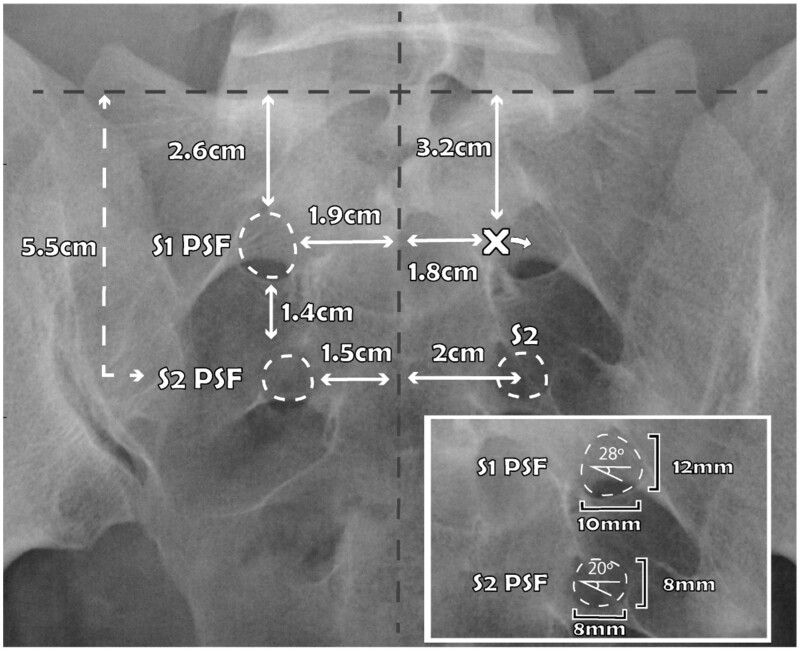
Sacral measurements under fluoroscopy with the sacral endplate aligned to 35°. Gray dashed- lines represent the midline and the aligned sacral promontory. Measurements are relatively consistent across the population. The ‘X’ marks the target adjacent to the foramen to contact periosteum before walking laterally into the foramen. The inset box demonstrates the angle at which the dorsal root ganglion and nerve root exit the intervertebral foramen. PSF = posterior sacral foramen.

Initial identification of the PSF can be challenging, as it is smaller and may be superimposed on the ASF, and additional variables can obscure its view under fluoroscopy including obesity, intestinal gas, osteoporosis, and instrumentation. This novel technique is designed to optimize foraminal access, reduce potential for neural insult, and allow the lead to lie in plane with the DRG and nerve root to minimize energy requirements. Sacral size and topography is relatively standard across the population, and this technique utilizes the consistent measurements from the sacral promontory to the S1 and S2 PSF [31]—see Figure 3.

**Figure 4.**
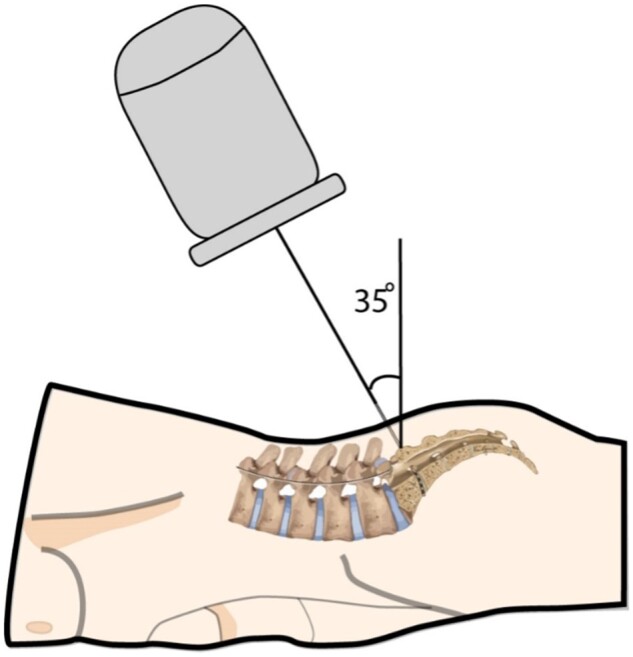
Fluoroscopic set up for visualization.

After sterile prep and drape, AP and lateral fluoroscopic evaluation ensure understanding of the midline, the sacral endplate, and even potential S1 or S2 interlaminar spaces that could create risk for inadvertent canal entry and dural puncture. Measurements are taken from the aligned sacral promontory. To compensate for the lumbosacral angle and sacrum convexity, align the sacral promontory with an approximately 35° craniocaudal angulation of the fluoroscope—see Figure 4. This also aligns the trajectory through the PSF and ASF. Using a skin marker, draw a line over the sacral promontory and another over the aligned midline sacral crest.

### S1 PSF Access

The initial target is the bony sacrum at the medial border of the PSF, which is 3.2 cm from the sacral promontory and 1.8 cm from the midline. Begin by confirming foraminal access with a 22-gauge Quincke ‘finder’ needle. This can limit the potential trauma and discomfort with repeated attempts using the 14-gauge DRG-S Tuohy needle. After contacting periosteum with the finder needle at the medial border, redirect the needle laterally and enter the PSF. The medial portion of the PSF may be slightly obscured by the intermediate crest (fused facet joint of S1). Confirm needle placement within the PSF using the lateral view. Avoid injecting local anesthetic at or beyond the PSF ligaments with the finder needle.

After locating the foramen, remove the 22-gauge needle and follow its tract with the Tuohy needle. Again, contact periosteum medially, redirect the needle laterally, and stop as the needle penetrates the PSF ligaments. At this point do not advance the Tuohy needle further. With the goal of minimizing inadvertent instrumentation of the ASF, check the lateral fluoroscopic image to confirm the Tuohy needle tip is at or just beyond the posterior wall of the sacral canal. The medial-to-lateral PSF entry allows the lead to run along the DRG and nerve root rather than colliding with it, optimizing available contacts for stimulation while potentially decreasing energy requirements.

After PSF entry, rotate the bevel of the Tuohy inferolateral, along the axis of the nerve root. With the introducer sheath preloaded with the DRG-S lead, advance the introducer sheath into the Tuohy needle until the first indicator line on the sheath reaches the hub of the needle, thereby keeping the sheath within the bevel. Attempt to pass the lead through the ASF without advancing the introducer sheath. This is typically easy, considering the large diameter of the S1 foramen. Pass the lead anteriorly until the distal contact nears the anterior sacral body. If unable to advance the lead, consider using the introducer wire from the kit to find a path that the lead can follow, similar to the thoracolumbar DRG-S placement technique. Gentle advancement of lead, introducer wire, or introducer sheath into the foramen is imperative to avoid irritation of the DRG. If unable to pass, consider repositioning the Tuohy needle rather than repeated failed attempts.

### Sacral Loop Placement

**Figure 5.**
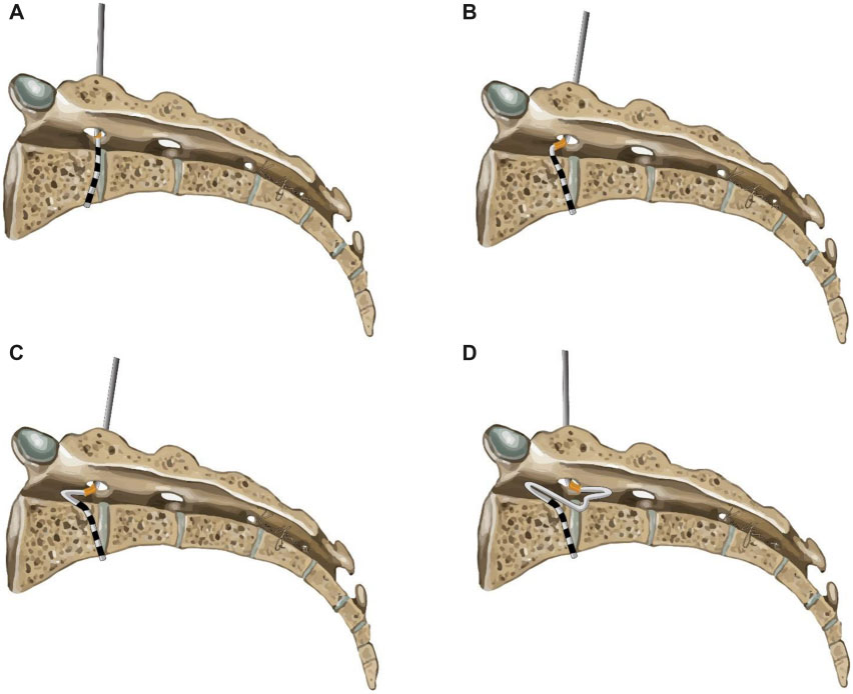
Sacral loop placement. (**A**) After the Tuohy needle passes the posterior sacral foramen, the lead is advanced without advancing the needle or introducer sheath further so the distal contact is at the level of the anterior sacral border. (**B**) The Tuohy needle with the sheath within the hub is rotated cephalad and the introducer sheath is advanced so it protrudes slightly from the hub. (**C**) Once the lead bends slightly, retract the stylet and advance the lead slowly. (**D**) Once the superior loop formed, retract sheath, rotate the needle caudally, and repeat the process.

With the distal contact near the anterior border of the sacrum, retract the lead stylet 5–7 cm, and with the introducer sheath within the bevel of the Tuohy, rotate the sheath and bevel rostrally—see Figure 5. To form the superior loop, lever the Tuohy caudad and advance the introducer sheath several millimeters rostrally, so the tip of the introducer sheath appears in the cephalad direction on fluoroscopy. Then, slowly advance the introducer sheath and lead rostrally in the sacral canal. Once the lead bows in the rostral direction, advance the lead.

Once the cephalad loop is created, retract the introducer sheath back into the Tuohy needle, rotate both the Tuohy and introducer sheath dorsally, and advance the lead. The lead may advance slightly into the S1 foramen, which may be acceptable if unable to correct, given the large foraminal diameter. This process can be repeated as needed.

Properly placed sacral canal loops are resilient to migration; however, placement can be challenging, especially at lower sacral foramina. If unable to place intracanal loops, an alternative is placement of tension loops external to the sacral canal, dorsal to the posterior sacral wall. Extra-canalicular loop placement requires the introducer sheath to direct the lead during loop placement. Attention is required to limit lead retraction and kinking of the sheath or lead secondary to excessive pressure on either component. Additional pressure is sometimes needed to find a potential track on the posterior sacral surface or in the overlying soft tissue.

### Limitations

Thin patients might not be candidates for the procedure due to the paucity of tissue overlying the sacral foramen.

## Conclusion

A heightened and organized understanding of sacral anatomy could potentially lead to a more effective and safe method of DRG-S lead placement. Alignment of the lumbosacral prominence and utilization of the measurement-based approach as described in this manuscript allows a standardized access, even when visualization is suboptimal. The medial-to-lateral needle trajectory is designed to limit interaction with the sensitive neural structures and allows for a more parallel orientation of the lead to the DRG and nerve root, potentially improving efficacy while lowering energy requirements.

## Authors’ Contributions

All authors contributed to the development of the technique and writing sections of the manuscript or provided critical reviews and substantive editing. K.B.C. served as primary author and project organizer.

## Notes

Funding sources: No funding was received for this work.

Disclosures: Jan Willem Kallewaard is on the advisory board Abbott, Saluda, Nevro, Boston Scientific. David M. Dickerson is a consultant for SPR therapeutics, Vertos medical, Abbott, Pfizer, Myovant, and Nalu. Jonathan M. Hagedorn is a consultant for Abbott, Boston Scientific, Medtronic, Nevro, and Saluda. Kiran V. Patel is a consultant for Abbott, Boston Scientific, and Averitas. Timothy R. Deer is a consultant for Abbott, Boston Scientific, Medtronic, Saluda, Nalu. Funded research from Abbott, Boston Scientific and Saluda. Soriaya Motivala is a consultant for Medtronic. Kenneth B. Chapman, Noud van Helmond, and Kris C. Vissers have nothing to disclose.

## References

[1] Vancamp T , Levy RM, Peña I, Pajuelo A. Relevant anatomy, morphology, and implantation techniques of the dorsal root ganglia at the lumbar levels. Neuromodulation 2017;20(7):690–702. 28895256 10.1111/ner.12651

[2] Chapman KB , Mogilner AY, Yang AH, et al Lead migration and fracture rate in dorsal root ganglion stimulation using anchoring and non-anchoring techniques: A multicenter pooled data analysis. Pain Pract 2021;21(8):859–70. 34145740 10.1111/papr.13052

[3] Chapman KB , Spiegel MA, Dickerson DM, et al A paramedian approach for dorsal root ganglion stimulation placement developed to limit lead migration and fracture. Pain Pract 2021;21(8):991–1000. 34328256 10.1111/papr.13063

[4] Rowe J. St. Jude Medical Dorsal Root Ganglion (DRG) Stimulator Procedure and The Rowe S1/S2 Placement Technique, 1–4. Available at: https://spinalcordstim.com (accessed January 21, 2022).

[5] Deer TR , Pope JE, Lamer TJ, et al The neuromodulation appropriateness consensus committee on best practices for dorsal root ganglion stimulation. Neuromodulation 2019;22(1):1–35. 30246899 10.1111/ner.12845

[6] Skaribas IM , Peccora C, Skaribas E. Single S1 dorsal root ganglia stimulation for intractable complex regional pain syndrome foot pain after lumbar spine surgery: A case series. Neuromodulation 2019;22(1):101–7. 29701900 10.1111/ner.12780

[7] Chapman KB , van Roosendaal BK, van Helmond N, Yousef TA. Unilateral dorsal root ganglion stimulation lead placement with resolution of bilateral lower extremity symptoms in diabetic peripheral neuropathy: A case report. Cureus 2020;12(9):e10735. 33145140 10.7759/cureus.10735 PMC7599049

[8] Chapman KB , Roosendaal B‐K, Yousef TA, Vissers KC, Helmond N. Dorsal root ganglion stimulation normalizes measures of pain processing in patients with chronic low back pain: A prospective pilot study using quantitative sensory testing. Pain Pract 2021;21(5):568–77. 33369117 10.1111/papr.12992

[9] Groenen PS , van Helmond N, Chapman KB. Chemotherapy-induced peripheral neuropathy treated with dorsal root ganglion stimulation. Pain Med 2019;20(4):857–9. 30412243 10.1093/pm/pny209

[10] Deer TR , Levy RM, Kramer J, et al Dorsal root ganglion stimulation yielded higher treatment success rate for complex regional pain syndrome and causalgia at 3 and 12 months. Pain 2017;158(4):669–81. 28030470 10.1097/j.pain.0000000000000814 PMC5359787

[11] Kobayashi S , Yoshizawa H, Yamada S. Pathology of lumbar nerve root compression Part 1: Intraradicular inflammatory changes induced by mechanical compression. J Orthop Res 2004;22(1):170–9. 14656677 10.1016/S0736-0266(03)00131-1

[12] Lin XY , Yang J, Li HM, Hu SJ, Xing JL. Dorsal root ganglion compression as an animal model of sciatica and low back pain. Neurosci Bull 2012;28(5):618–30. 23054639 10.1007/s12264-012-1276-9 PMC5561928

[13] Hagedorn JM , Deer TR, Falowski SM, et al An observational study of intraoperative neuromonitoring as a safety mechanism in placement of percutaneous dorsal root ganglion stimulation and spinal cord stimulation systems. J Pain Res 2020;13:3349–53. 33324094 10.2147/JPR.S289416 PMC7733403

[14] Horan M , Jacobsen AH, Scherer C, et al Complications and effects of dorsal root ganglion stimulation in the treatment of chronic neuropathic pain: A nationwide cohort study in Denmark. Neuromodulation 2021;24(4):729–37. 32539189 10.1111/ner.13171

[15] Khan Z , Shankar H. Lumbar 5 nerve root injury following dorsal root ganglion stimulator lead placement. Neuromodulation 2020;23(2):258–9. 30844113 10.1111/ner.12945

[16] Yuan AS , Almodovar JL, Erekson E. Neurologic injury after sacral neuromodulation. Female Pelvic Med Reconstr Surg 2019;25(2):e45–6. 30730349 10.1097/SPV.0000000000000701

[17] Swinn M , Schott G, Oliver S, Kitchen N, Fowler C. Leg pain after sacral neuromodulation: Anatomical considerations. BJU Int 1999;84(9):1113–5. 10571653 10.1046/j.1464-410x.1999.00419.x

[18] Falowski S , Pope JE, Raza A. Early US experience with stimulation of the dorsal root ganglia for the treatment of peripheral neuropathy in the lower extremities: A multicenter retrospective case series. Neuromodulation 2019;22(1):96–100. 30264870 10.1111/ner.12860

[19] Pal GP. Weight transmission through the sacrum in man. J Anat 1989;162:9–17. 2808126 PMC1256432

[20] Abitbol MM. Evolution of the lumbosacral angle. Am J Phys Anthropol 1987;72(3):361–72. 3107397 10.1002/ajpa.1330720309

[21] Saluja S , Agarwal S, Tuli A, Raheja S, Tigga SR, Paul S. Morphometric analysis of the Sacrum and its surgical implications. J Clin Diagnostic Res 2018;12(6):AC01–6.

[22] Arman C , Naderi S, Kiray A, et al The human sacrum and safe approaches for screw placement. J Clin Neurosci 2009;16(8):1046–9. 19442524 10.1016/j.jocn.2008.07.081

[23] Xu R , Ebraheim NA, Robke J, Huntoon M, Yeasting RA. Radiologic and anatomic evaluation of the anterior sacral foramens and nerve grooves. Spine 1996;21(4):407–10. 8658241 10.1097/00007632-199602150-00001

[24] Whelan MA , Gold P. R. Computed tomography of the sacrum. I. Normal anatomy. Am J Neuroradiol 1982;3(5):547–54. 10.2214/ajr.139.6.1183 6983265

[25] Cha YD , Choi JK, Yang CW, Lim HK, Heo GA, Kim BG. Relationship between first dorsal sacral foramen and lumbar facet joint connecting line in South Korea populations. Med (United States) 2017;96(29):e7544. 10.1097/MD.0000000000007544 PMC5521914 28723774

[26] Esses SI , Botsford DJ, Huler RJ, Rauschning W. Surgical anatomy of the sacrum: A guide for rational screw fixation. Spine 1991;16(suppl 6):S283–8. 1862426 10.1097/00007632-199106001-00021

[27] Hasan S , Shanahan D, Pridie A, Neal D. Surface localization of sacral foramina for neuromodulation of bladder function. An anatomical study. Eur Urol 1996;29(1):90–8. 8821698 10.1159/000473725

[28] Ebraheim NA , Lu J. Morphometric evaluation of the sacral dorsal root ganglia: A cadaveric study. Surg Radiol Anat 1998;20(2):105–8. 9658528

[29] Kang RA , Sim WS, Choi JW, et al Comparison between anteroposterior and oblique “Scotty dog” approach during S1 transforaminal epidural steroid injection: A randomized controlled trial. Medicine (Baltimore) 2020;99(43):e22895. 33120838 10.1097/MD.0000000000022895 PMC7581171

[30] Fish DE , Lee PC, Marcus DB. The S1 “Scotty Dog”: Report of a technique for S1 transforaminal epidural steroid injection. Arch Phys Med Rehabil 2007;88(12):1730–3. 18047894 10.1016/j.apmr.2007.07.041

[31] Arora S , Verma M, Kaur S, Chhabra S, Jain P. Measurement of sacral parameters of surgical importance in North Indian population. J Clin Diagnostic Res 2018;12(2):AC05–9.

